# P-1781. Machine Learning Model for Differentiating Pneumocystis jirovecii Pneumonia from Colonization and Analyzing Mortality Risk in Non-HIV Patients Using BALF Metagenomic Sequencing

**DOI:** 10.1093/ofid/ofaf695.1950

**Published:** 2026-01-11

**Authors:** Yuhui Chen, Meng Li, Xinai Gan, Yutong Wang, Pazilaiti Tuohuti, Yongzhao Zhou, Ting Niu

**Affiliations:** Department of Hematology, Institute of Hematology, West China Hospital, Sichuan University, Chengdu, Sichuan, China (People's Republic); West China Hospital, Sichuan University, Chengdu, Sichuan, China; West China Hospital, Sichuan University, Chengdu, Sichuan, China; West China Hospital, Sichuan University, Chengdu, Sichuan, China; West China Hospital, Sichuan University, Chengdu, Sichuan, China; West China Hospital, Sichuan University, Chengdu, Sichuan, China; West China Hospital, Sichuan University, Chengdu, Sichuan, China

## Abstract

**Background:**

Diagnosing Pneumocystis jirovecii infection in non-HIV patients is challenging, distinguishing PJP from colonization, and treatment options are limited.

**Figure:**
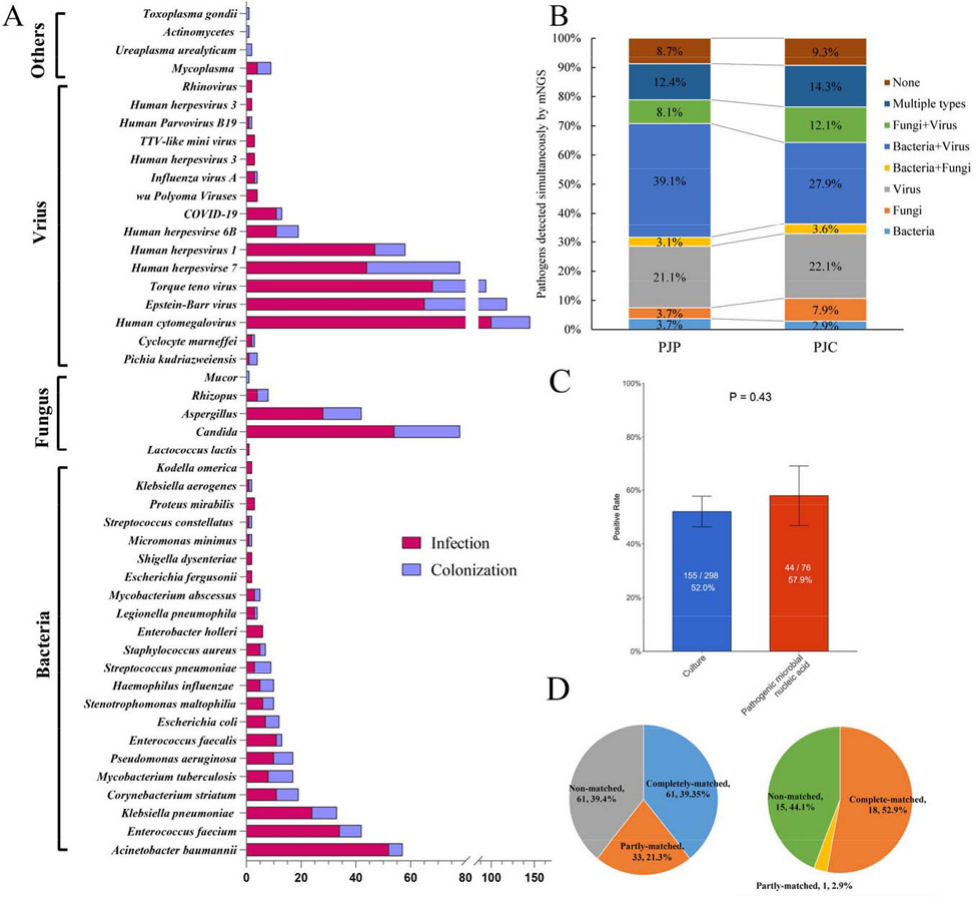
(A) Distribution of pathogens identified by mNGS in BALF samples. Red bars represent the infected group; light blue bars represent the colonized group. (B) Distribution of microorganism types detected by mNGS in BALF samples; (C) Comparison of positivity rates between culture and pathogenic PCR methods; (D) Concordance between mNGS and conventional methods: culture (left) and PCR (right). Selection of high-risk independent predictor variables by LASSO regression and construction and validation of clinical prediction model for differentiating PJP and PJC in patients undergoing mNGS using BALF by XGBoost, Logistic Regression, Random Forest, Support Vector Machine (SVM), and Neural Network.

**Figure:**
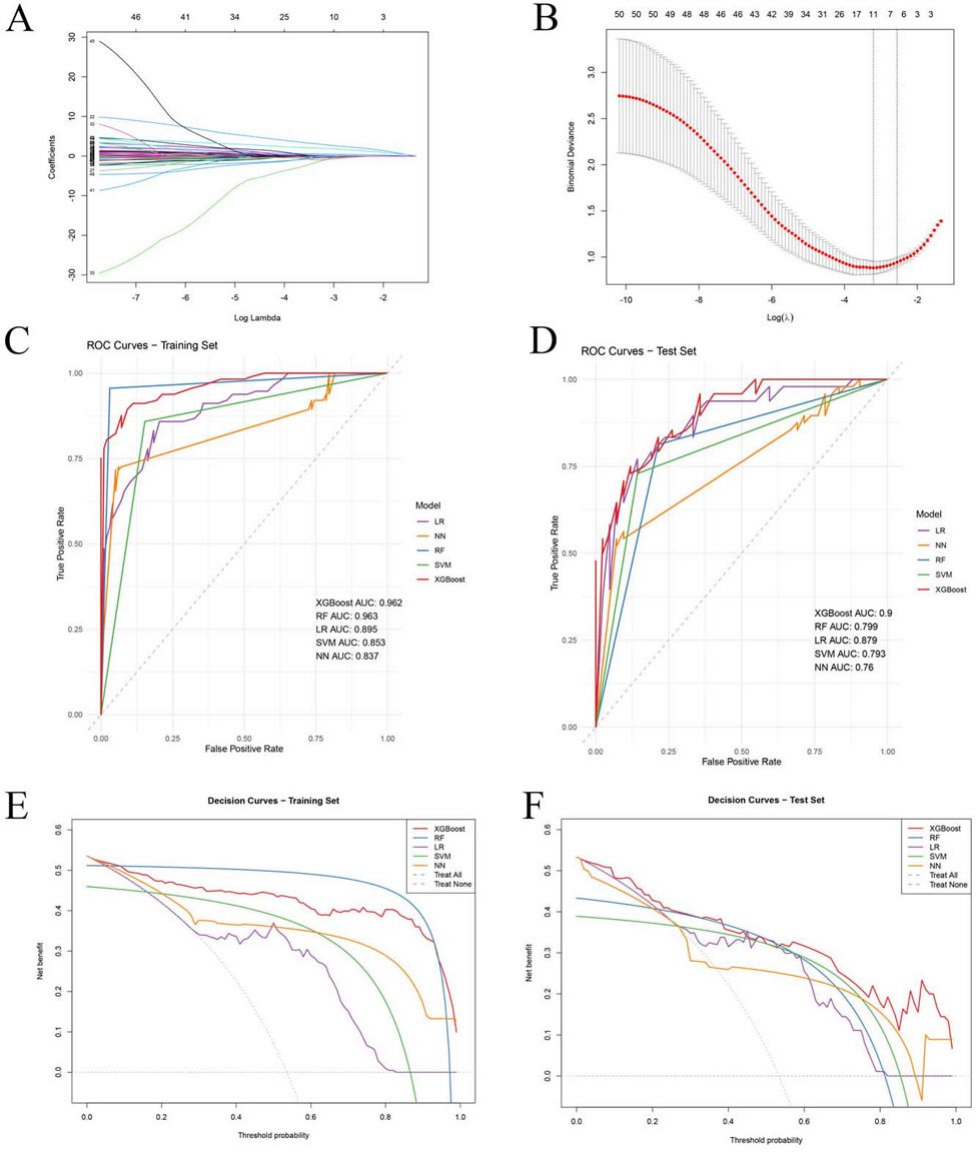
(A) LASSO Coefficient Path Plot for P. jirovecii Infection Prediction Model. (B) Cross-Validated Mean Squared Error for LASSO Model Selection. (C) ROC curves of the five prediction models in in training set. (D) ROC curves of the five prediction models in in test set. (E) Decision curve analysis (DCA) of the five prediction models in training set. (F) Decision curve analysis (DCA) of the five prediction models in test set.

**Methods:**

We conducted a retrospective analysis of 301 non-HIV patients from August 2020 to February 2024, who had *P. jirovecii* detected in bronchoalveolar lavage fluid (BALF) using metagenomic next-generation sequencing (mNGS). Based on clinical criteria, patients were categorized into PJP (n=161) and *P. jirovecii* colonization (PJC, n=140) groups. We assessed five machine learning algorithms, selecting XGBoost as the final predictive model, and applied SHapley Additive exPlanations (SHAP) analysis for interpretability. Additionally, we identified 28-day mortality risk factors in PJP patients and performed subgroup survival analyses.

**Figure:**
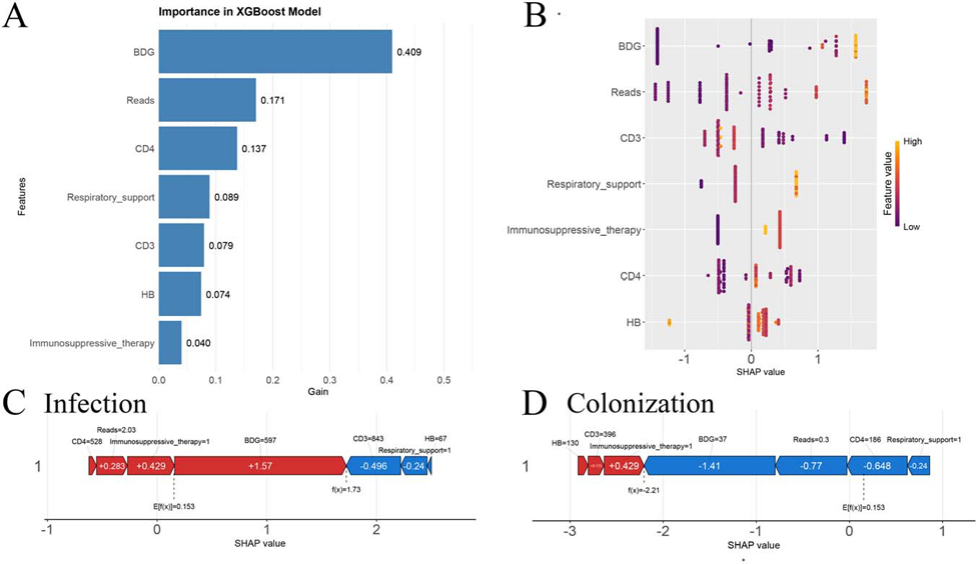
XGBoost model interpretation and SHAP analysis for PJP prediction. (A) Feature importance ranking derived from the XGBoost model. Bars represent the relative importance of each predictor variable in the model, based on their contribution to improving the model's performance. (B) SHAP summary plot for the test set. Each point represents a sample, with colors indicating feature values (purple for low, yellow for high). The x-axis shows the SHAP value impact on the model output, while features are ordered by their overall importance. (C) SHAP Individual Force Plot for a representative case predicted as PJP, illustrating how each feature contributes to the model's prediction; (D) SHAP individual force plot for a representative case predicted as PJC, showing the impact of each feature on the model's prediction for colonization.

**Figure:**
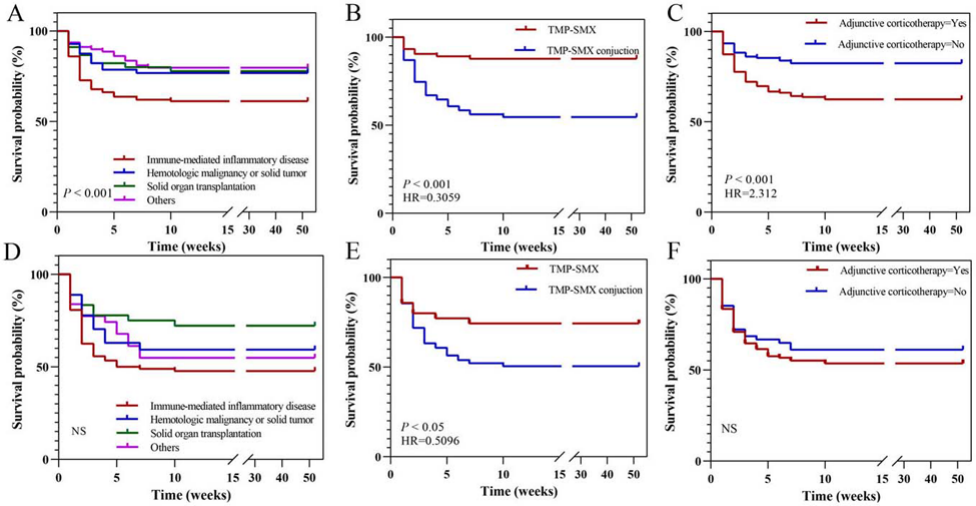
Patient Survival Outcomes. (A) Survival rates across four different disease etiologies. (B) Survival rates comparing corticosteroid users vs. non-users with PaO2/FiO2 > 300 mmHg. (C) Survival rates comparing corticosteroid users vs. non-users with PaO2/FiO2 < 300 mmHg. (D) Survival rates in groups with multiple, dual, single, and no co-infections identified by mNGS. (E) Survival rates within patients with dual-type co-infections. (F) Survival rates within patients with single-type co-infections.

**Results:**

The XGBoost model achieved an area under the curve (AUC) of 0.9000 in distinguishing PJP from PJC, outperforming all other tested algorithms. Key predictors included BDG levels, log-transformed reads for *P. jirovecii*, CD4^+^ T cell count, and respiratory support. Significant risk factors for 28-day mortality in PJP patients included the use of decreased PaO_2_/FiO_2_ ratios (final OR: 0.98, *P* < 0.001), lower platelet counts (final OR: 0.98, *P* =0.057), lower CD3^+^ (final OR: 0.99, *P* = 0.034), as was a lower CD4^+^ T cell count (final OR: 0.98, *P* = 0.023). Patients with immune-mediated inflammatory diseases experienced the lowest survival rates. The use of corticosteroids did not enhance survival, regardless of patients having good or poor oxygenation status. Co-infections, particularly those with multiple pathogens, were associated with the most adverse outcomes, with "bacterial + viral" co-infections posing the greatest risk among dual pathogens, and bacterial infections being the most detrimental in single-pathogen scenarios.

**Conclusion:**

This study highlights the utility of mNGS in detecting *P. jirovecii* and the effectiveness of the XGBoost model in differentiating PJP from PJC. Patients with immune-mediated diseases and co-infections exhibit poorer outcomes. Corticosteroids did not enhance survival.

**Disclosures:**

All Authors: No reported disclosures

